# Exploring cancer register data to find risk factors for recurrence of breast cancer – application of Canonical Correlation Analysis

**DOI:** 10.1186/1472-6947-5-29

**Published:** 2005-08-22

**Authors:** Amir R Razavi, Hans Gill, Olle Stål, Marie Sundquist, Sten Thorstenson, Hans Åhlfeldt, Nosrat Shahsavar, the South-East Swedish Breast Cancer Study Group

**Affiliations:** 1 Department of Biomedical Engineering, Division of Medical Informatics, Linköping University, Sweden; 2 Department of Biomedicine and Surgery, Division of Oncology, Linköping University, Sweden; 3 Department of Surgery, County Hospital, Kalmar, Sweden; 4 Department of Pathology, County Hospital, Kalmar, Sweden; 5 Oncology Centre, University Hospital, Linköping University, Sweden

## Abstract

**Background:**

A common approach in exploring register data is to find relationships between outcomes and predictors by using multiple regression analysis (MRA). If there is more than one outcome variable, the analysis must then be repeated, and the results combined in some arbitrary fashion. In contrast, Canonical Correlation Analysis (CCA) has the ability to analyze multiple outcomes at the same time.

One essential outcome after breast cancer treatment is recurrence of the disease. It is important to understand the relationship between different predictors and recurrence, including the time interval until recurrence. This study describes the application of CCA to find important predictors for two different outcomes for breast cancer patients, loco-regional recurrence and occurrence of distant metastasis and to decrease the number of variables in the sets of predictors and outcomes without decreasing the predictive strength of the model.

**Methods:**

Data for 637 malignant breast cancer patients admitted in the south-east region of Sweden were analyzed. By using CCA and looking at the structure coefficients (loadings), relationships between tumor specifications and the two outcomes during different time intervals were analyzed and a correlation model was built.

**Results:**

The analysis successfully detected known predictors for breast cancer recurrence during the first two years and distant metastasis 2–4 years after diagnosis. Nottingham Histologic Grading (NHG) was the most important predictor, while age of the patient at the time of diagnosis was not an important predictor.

**Conclusion:**

In cancer registers with high dimensionality, CCA can be used for identifying the importance of risk factors for breast cancer recurrence. This technique can result in a model ready for further processing by data mining methods through reducing the number of variables to important ones.

## Background

Breast cancer is the most common type of cancer diagnosed in women in Western countries. Sweden has had a high incidence of breast cancer for several decades, although mortality rates have been lower than in most other Western countries [1].

Breast cancer prognosis is influenced by many factors such as morphological and pathological tumor specifications and biological tumor markers. Studying these predictors and finding those of most importance can give clinicians better insight regarding the prognosis.

As a rule, data on cancer patients have been collected in all regions of Sweden since 1960. The data have been used for different purposes including epidemiological studies, monitoring and evaluating medical interventions, and finding risk factors for specific types of cancer [2-4]. A common approach in using data in registers for finding relationships between outcomes and predictors is to use multiple regression analysis (MRA). If the aim of the study is to determine the degree of importance of each predictor with more than one outcome variable, then the analysis has to be repeated, and the results must be combined in some arbitrary fashion.

In MRA, identification of important predictors is usually done by looking at the regression weights associated with each predictor. If the variables in the analysis are correlated among themselves (multicollinearity), it is then difficult to interpret the importance of the individual variables [5]. However, a seldom-used alternative approach is to compute loadings (structure coefficients) and use them as indicators of important predictors.

Hotelling (1936) developed Canonical Correlation Analysis (CCA) as a method for evaluating linear correlation between sets of variables [6]. The method allows investigation of the relationship between two sets of variables that can identify important variables in a set of multiple predictors and a set of multiple outcomes. Loadings and weights can be calculated by CCA with a module that is included in commercial statistical packages.

In the analysis of cancer recurrence, it is necessary to consider time as a fundamental factor, since different parameters are related to recurrence after a certain period of time. Some methods like Cox regression analysis are specially developed for handling events that occur during different times and when some cases are censored [7] (outcome not known when the study period ends).

In this study, CCA was used for identifying the importance of risk factors for breast cancer recurrence within specified time intervals. CCA was applied to data from breast cancer patients in the south-east region of Sweden.

## Methods

In this study, data from 637 female patients, mean age 59.5 years, were analyzed. The same patients as in the study by Sundquist et al. was used [8]. The aim of that study was to assess the applicability of histopathological grading as a prognostic index applied to a defined breast cancer population. Only patients without any sign of distant metastasis at the time of surgery were included in the analysis. Tumors with an invasive component of 2 millimetres or less in diameter were excluded from analysis because the small size did not permit proper grading in accordance with the protocol.

To obtain a more comprehensive dataset for this study, patient data were retrieved from three different registers, i.e. the regional breast cancer-, tumor markers- and cause of death registers.

### Predictors and outcomes

In order to answer important questions such as: "Which variables might be important predictors for recurrence of breast cancer? Is the time interval after diagnosis important? Is there a way to determine the importance of each predictor when there is more than one type of recurrence?" two sets of predictors and outcomes (see Table 1) were selected by consulting and collaborating with oncologists and studying the literature in the domain.

**Table 1:** List of variables in both sets

**Predictor Set**	**Outcome Set ^‡^**
Age	DM, first two years
Tumor location	DM, 2–4 years
Side	DM, more than 4 years
Tumor size *	LRR, first two years
LN involvement *	LRR, 2–4 years
LN involvement ^†^	LRR, more than 4 years
Periglandular growth *	
NHG	
Multiple tumors *	
Estrogen receptor	
Progesterone receptor	
S-phase fraction	
DNA index	
DNA ploidy	

Abbreviations: LN: lymph node, NHG: Nottingham Histologic Grade, DM: Distant Metastasis, LRR: Loco-regional Recurrence

* from pathology report, ^† ^N0: Not palpable LN metastasis, ^‡ ^all periods are time after diagnosis.

Age of the patient and variables regarding tumor specifications based on pathology reports, physical examination and tumor markers were selected as predictors. Two variables in the outcome set, distant metastasis and loco-regional recurrence were observed at different time intervals after diagnosis.

### Data preprocessing

After retrieving information in different registers about selected predictors and outcomes (Table 1) for 637 patients, the raw data were transformed and converted as illustrated in Table 2.

**Table 2:** Transformation rules and the study population characteristics

**Variable**	**Categories**	**Coded as**	**n**
Age	>50 years	0	177
	≤ 50 years	1	459
Tumor location	(not) Superior medial	(0)1	144
	(not) Inferior medial	(0)1	70
	(not) Superior lateral	(0)1	368
	(not) Inferior lateral	(0)1	112
	(not) Nipple area	(0)1	58
Side	Left	0	315
	Right	1	322
Tumor Size	≤ 20 mm	0	233
	>20 mm	1	404
LN involvement	No LN involvement	0	373
	Positive LN involvement	1	260
LN involvement (N0)	No palpable LN	0	100
	Palpable and/or fixed LNs	1	533
Periglandular growth	Absence of growth	0	515
	Presence of growth	1	122
Nottingham Histologic Grade	I	1	145
	II	2	228
	III	3	264
Multiple tumors	Absence of multiple tumors	0	502
	Presence of multiple tumors	1	134
Estrogen receptor	≥ 0.3 fmol/mg	0	181
	<0.3 fmol/mg	1	456
Progesterone receptor	≥ 0.3 fmol/mg	0	232
	<0.3 fmol/mg	1	405
S-phase fraction	<10%	0	439
	≥ 10%	1	198
DNA index (DI)	0.9 ≤ DI and DI < 1.3	0	345
	0.9 > DI or DI ≥ 1.3	1	292
DNA ploidy	DNA diploidy or tetraploidy	0	368
	DNA aneuploid	1	269

For some variables such as LN involvement, periglandular growth and multiple tumors, dichotomization was done based on their presence or absence in the patients. Other variables such as tumor location, side, Nottingham Histologic Grade and DNA ploidy were already categorical. The remaining variables, i.e. age, tumor size, estrogen and progesterone receptors, S-phase fraction and DNA index, were transformed from continuous to dichotomous variables (Table 2).

Missing values were substituted using the Expectation maximization (EM) algorithm [9]. This algorithm is a parameter estimation method, which falls within the general framework of maximum likelihood estimation and is an iterative optimization algorithm.

### Canonical Correlation Analysis

Because the outcome set consists of several variables, CCA, which is a technique for analyzing the relationship between two sets of variables, was performed.

The fundamental principle behind CCA is the creation of a number of canonical solutions [5], each consisting of a linear combination of one set of variables, which has the form:

*U_i _= a_1_(predictor_1_) *+ *a_2_(predictor_2_) *+ ... + *a_m_(predictor_m_)*

and a linear combination of the other set of variables, which has the form:

*V_i _= b_1_(outcome_1_) *+ *b_2_(outcome_2_) *+ ... + *b_n_(outcome_n_)*

The goal is to determine the coefficients (a's and b's) that maximize the correlation between canonical variates U_i _and V_i_. The number of solutions is equal to the number of variables in the smaller set. The first canonical correlation is the highest possible correlation between any linear combination of the variables in the predictor set and any linear combination of the variables in the outcome set.

A way of interpreting the canonical solutions is to look at the correlations between the canonical variates and the variables in each set. These correlations are called structure coefficients or loadings. The logic here is that variables that are highly correlated with a canonical variate have more in common with it and they should be considered more important when deriving a meaningful interpretation of the related canonical variate. This way of interpreting canonical variates is identical to the interpretation of factors in factor analysis [10]. The criterion for choosing the important variables in each canonical variate is the structure coefficients (loadings). As a rule of thumb for meaningful loadings, an absolute value equal to or greater than 0.3 is often used [11,12].

Significance of the canonical correlations was tested with randomization tests, and robustness of the estimates of the loadings was tested with bootstrapping [13].

SPSS version 11 [14] was used for data transformation and replacing missing values. For running CCA, the CANCORR macro, a part of the Advanced Statistics module of SPSS, was used. Tests of significance for canonical correlations and bootstrapping were done using MATLAB Ver 6.5 [15].

## Results

Table 2 shows the study population characteristics. The relationships between predictors and outcomes (Table 1) were analyzed by CCA, which generated six solutions, equal to the number of outcome variables.

For the first solution, the canonical correlation coefficient (rc) was equal to 0.547 with the p value ≤ .001.

Table 3 gives the individual Structure Coefficients (loadings) between the tumor specifications and their canonical variate (U1) and between the recurrences of breast cancer and their canonical variate (V1) for the first solution. Important variables (absolute value of loading ≥ 0.3) are shown in bold type in the table. Other variables with lower loadings are not considered important for the interpretation.

**Table 3:** Canonical Structure Matrix for Predictor and outcome Variates

**Predictor Set**	**U_1_**	**Outcome Set^‡^**	**V_1_**
Age	.223	**DM, first two years**	**.837**
Tumor location		**DM, 2–4 years**	**.332**
Superior medial	.138	DM, more than 4 years	.193
Inferior medial	.159	**LRR, first two years**	**.486**
Superior lateral	-.056	LRR, 2–4 years	-.030
Inferior lateral	.155	LRR, more than 4 years	-.013
Nipple area	.160		
Side	-.017		
Tumor size *	**.432**		
LN involvement *	**.567**		
**LN involvement (N0) ^†^**	**.580**		
**NHG**	**.697**		
Perigland growth *	**.566**		
Multiple tumors *	.110		
**Estrogen receptor**	**.370**		
**Progesterone receptor**	**.365**		
**S-phase fraction**	**.629**		
**DNA index**	**.325**		
**DNA ploidy**	**.342**		

Abbreviations: LN: lymph node, NHG: Nottingham Histologic Grade, DM: Distant Metastasis, LRR: Loco-regional Recurrence

* from pathology report, ^† ^N0: Not palpable LN metastasis, ^‡ ^all periods are time after diagnosis.

If the signs in the sets are the same, then if one increases the other also increases, and vice versa.

When displaying loadings, signs help to identify the character of the relationship between variables in the predictor and outcome sets. If both have the same sign then they change in the same direction; if one increases then the other will also increase, and vice versa.

**Figure 1:**
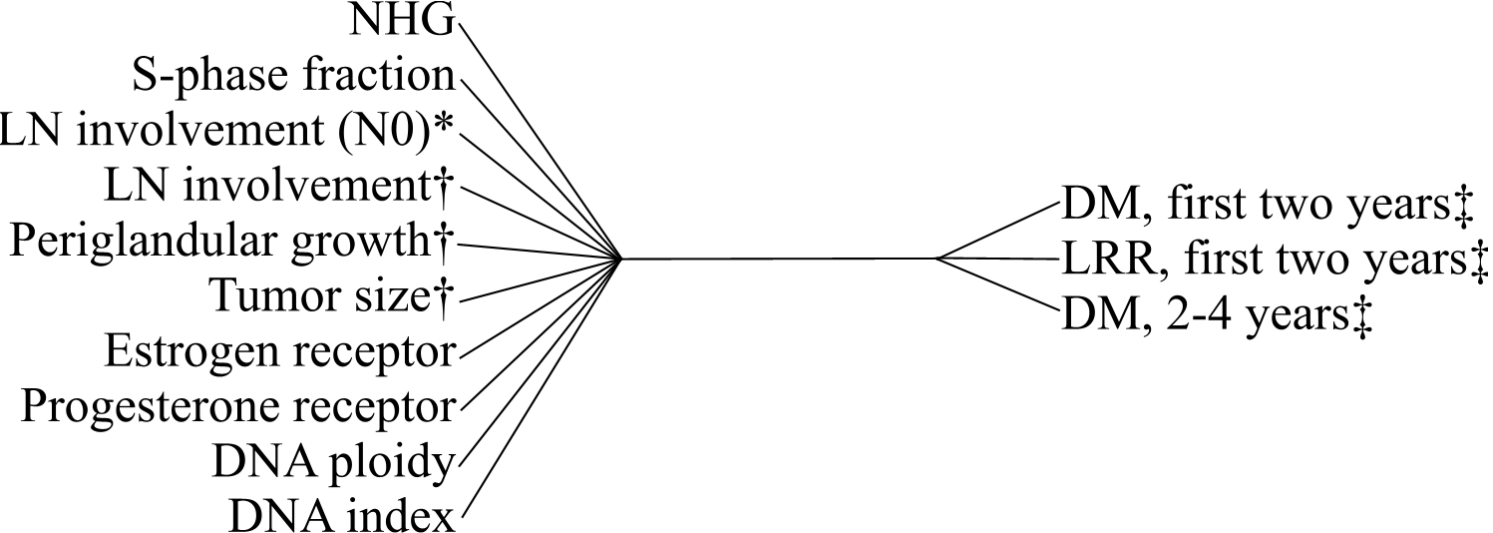
**The first canonical correlation solution. Variables are sorted by the absolute value of their loadings. **Abbreviations: LN: lymph node, DM: Distant Metastasis, LRR: Loco-regional Recurrence. * N0: Not palpable LN metastasis, † from pathology report, ‡ all periods are time after diagnosis. If the signs in the sets are the same, then if one increases the other also increases, and vice versa.

The first solution is illustrated in Figure 1. The variables in both sets are arranged by the absolute values of the loadings, which show their importance within each canonical variate. By considering loadings and signs in the first solution, patients with higher NHG, higher S-phase fraction, presence of lymph node involvement (based on pathology reports and physical examination), presence of periglandular growth, larger tumor size, negative estrogen and progesterone receptor status, DNA aneuploidy and abnormal DNA index are associated with an increased risk for distant metastasis (DM) and loco-regional recurrence (LRR) during the first two years after diagnosis of breast cancer, and DM 2–4 years after diagnosis. In the predictor set, age, existence of multiple malignant tumors, and side and tumor location did not get meaningful loadings so they are assumed to be unimportant as predictors for recurrence of the disease.

## Discussion

The use of CCA loadings facilitates the detection of important predictors, particularly when there are many variables in the dataset and there are high correlations among those variables. The ability to find important predictors when we are not restricted to using just one outcome variable means that we have a more general tool for analyzing the data. CCA can be used for the purpose of dimension reduction prior to a data mining step for knowledge discovery in databases. Using exploratory multivariate statistics such as CCA, the effective number of variables can be reduced while preserving the information content.

### Methodological consideration

In the present study we show that CCA provides overall associations between tumor specifications and breast cancer outcomes derived from the datasets in the registers.

If the interpretation is based on the level of loadings, high correlations between variables do not disturb the interpretation. This is in contrast to analyzing variables based only on the significance of the weights. Cooley and Lohnes suggest examining loadings as a better criterion for finding important predictors, especially when the goal is to determine which variables relate most strongly to the linear composite that best predicts the outcomes [16].

Since MRA is in fact a special case of CCA when the outcome set consists of just one variable, both loadings and weights can also be calculated for MRA, but in standard statistics software only the weights are calculated [17].

The number of cases studied is important in CCA. If there are too few cases, the results will not be reliable. Barcikowski and Stevens, in a Monte Carlo study on the stability of the coefficients and the correlations in canonical correlation analysis, found that a ratio of about 20:1 between the number of records and the number of variables is sufficient for accurate interpretation [18,19]. In our study, the ratio was about 25:1, which means that we had a sufficient number of cases.

CCA is not commonly used in medicine. Its limited use may be due to a lack of familiarity with the method and complexity in the calculations, but CCA is now included in statistics software packages such as SPSS and SAS.

The significance of the analysis and the robustness of the results were successfully tested with randomization tests and bootstrapping.

The results support the correctness and validity of the registers that were used, since many of the important variables were confirmed based on the knowledge and experience of oncology specialists.

### Medical interpretation

In the first solution (Figure 1), Nottingham Histologic Grading (NHG) got the highest loading. This grading technique involves semiquantitative evaluation of three morphological features and a numerical scoring system. This score is also a well-known and important predictor for the prognosis of breast cancer [20].

The S-phase fraction is a measure of the percentage of cells in cancer cells that are in the phase of the cell cycle during which DNA is synthesized. Other studies have shown that higher fractions are generally associated with poorer overall survival [21]. In our study, S-phase fraction got second place among predictors.

Examining lymph node involvement is essential in assessing the probability of breast cancer recurrence. In this study, variables showing nodal involvement are important and got third and fourth place among predictors. The overall survival of patients has been shown to decrease as nodal involvement increases [22].

Periglandular growth of the malignant tumor [23], size of the tumor [8], receptors for estrogen and progesterone [24], DNA ploidy and DNA index [25,26] were also found to be important predictors in the present study.

Some studies indicate that age is an important factor [27] and the younger the age of the patient, the poorer the prognosis for the disease. However, in this study, age, used either as a continuous variable or as a categorical one, did not get any meaningful loading.

There were other variables such as side and location of the tumor that based on their loadings were not considered important predictors (Table 3).

Looking at the outcome set for the first solution, we see that the important predictors are related to the occurrence of DM during the first four years and LRR during the first two years after diagnosis.

In this study, we have presented the CCA method for exploring registered data using a proposed model for predictors and multiple outcome variables. CCA can analyse different models including combination of predictors or further predictors such as genetic risk factors, node ratio or family history of breast cancer. Flexibility in creating different models can also cover several outcomes in different time intervals. However, construction of a model is an important step because predictors and outcomes should be meaningful medically or epidemiologically as far as clinical decision making is concerned.

## Conclusion

CCA is suggested as an appropriate method when there are many variables in the input set and more than one variable in the output set. Applying CCA to the available dataset and reducing the number of variables to the important ones can promote further analysis in data mining methods. This can be assumed as the dimension reduction step in the whole process of knowledge discovery in the databases. By reducing the effort involved in manual interventions in analyzing data, CCA can also be helpful in real-time analytical processes.

We analyzed the relationship between tumor specifications and outcomes for breast cancer during different time intervals. The results of the main analysis successfully detected well known predictors for breast cancer recurrence in the input set. Nottingham Histologic Grade (NHG) was the most important prognostic variable in breast cancer patients. The next most important factors were S-phase fraction and nodal status.

## List of abbreviations

CCA: Canonical Correlation Analysis

MRA: Multiple Regression Analysis

NHG: Nottingham Histologic Grading

LN: Lymph Node

DM: Distant Metastasis

LRR: Loco-regional Recurrence

## Competing interests

The author(s) declare that they have no competing interests.

## Authors' contributions

This research is done as a part of ARR's PhD education under the supervision of NS with the HG, HÅ, OS as co-supervisors. Pathologic part of the study was done by MS and ST. The South-east Swedish breast cancer study group approves the project.

All authors read and approved the final manuscript.

## Pre-publication history

The pre-publication history for this paper can be accessed here:


